# Quantifying the health impacts of air pollution under a changing climate—a review of approaches and methodology

**DOI:** 10.1007/s00484-012-0625-8

**Published:** 2013-01-25

**Authors:** Sarunya Sujaritpong, Keith Dear, Martin Cope, Sean Walsh, Tord Kjellstrom

**Affiliations:** 1 National Centre for Epidemiology and Population Health, Canberra, ACT Australia; 2 Centre for Australian Weather and Climate Research, Melbourne, Victoria Australia; 3 EPA Victoria, Melbourne, Victoria Australia

**Keywords:** Air pollution, Climate change, Health, Projection, Methodology

## Abstract

Climate change has been predicted to affect future air quality, with inevitable consequences for health. Quantifying the health effects of air pollution under a changing climate is crucial to provide evidence for actions to safeguard future populations. In this paper, we review published methods for quantifying health impacts to identify optimal approaches and ways in which existing challenges facing this line of research can be addressed. Most studies have employed a simplified methodology, while only a few have reported sensitivity analyses to assess sources of uncertainty. The limited investigations that do exist suggest that examining the health risk estimates should particularly take into account the uncertainty associated with future air pollution emissions scenarios, concentration-response functions, and future population growth and age structures. Knowledge gaps identified for future research include future health impacts from extreme air pollution events, interactions between temperature and air pollution effects on public health under a changing climate, and how population adaptation and behavioural changes in a warmer climate may modify exposure to air pollution and health consequences.

**Electronic supplementary material:**

The online version of this article (doi:10.1007/s00484-012-0625-8) contains supplementary material, which is available to authorized users.

## Introduction

With increasing awareness of the threat posed to population health by climate change, there is demand from the public health community to quantitatively estimate the associated health burdens (World Health Organization 2009). Such quantification can help prioritise diseases and health outcomes, vulnerable groups, and affected populations so that appropriate policies and strategies can be developed. Rapid developments in climate science, particularly in the use of downscaling techniques, provide opportunities to investigate climate-related health consequences at the regional, state and city levels (Rosenthal et al. 2004).

The health impacts of air pollution are likely to be modified by climate change (World Health Organization 2003), due mainly to the exposure of populations to raised levels of air pollutants such as volatile organic compounds (VOCs), O_3_ and some components of secondary particles. The emission and production rates of these substances can be enhanced in a warmer climate (Hogrefe et al. 2005). Future projections of surface O_3_ and particulate matter (PM) have been undertaken more than other air pollutants because of their importance to public health (Ebi and McGregor 2008). Jacob and Winner (2009) summarized regional scale O_3_ projections and found that future surface O_3_ concentrations in the US and Europe could be increased by 1–10 ppb over the next century, particularly in polluted areas. On the other hand, future PM concentrations have been projected to vary with location. The authors attributed this variation to wide range of PM constituents and their different responses to changing meteorology. According to four studies conducted in US regions, precipitation was found to be an important determinate of future PM concentrations (Avise et al. 2009; Pye et al. 2009; Tagaris et al. 2007; Racherla and Adams 2006). Various sources of air pollution may respond differently to a future climate. For air pollution from natural sources, VOCs evaporated from vegetation may be increased with rising temperatures, as well as PM emitted from forest fires in areas projected to be drier (Flannigan et al. 2009). Similarly, changes in temperature can alter emissions from anthropogenic sources. For example, the reduced use of wood heaters in a temperate location can lead to a reduction of atmospheric PM concentrations for a warming climate trend (Cope et al. 2011b).

Despite the current importance of air pollution in determining disease burdens globally (Ostro 2004) and its sensitivity to climate change, only limited studies have attempted to quantify future health effects. This is partly due to the advanced and complex methods required in the quantification. Quantifying potential air pollution-related health effects requires air quality models (AQMs) to predict future air pollution levels based on climate model results, before they can be linked to health impacts functions. In addition, because of its interdisciplinary nature, such quantification demands collaboration from professionals in climate science, air quality and public health.

In this paper, we review methods that have been applied to quantify how future climate change will modify air pollution-related health effects. Past reviews focussed on study outcomes (Ebi and McGregor 2008; Kinney 2008; Barnett and Hansen 2009). In contrast, this review seeks to identify optimal approaches, and to suggest ways in which existing challenges can be addressed in order to attain improved health impact estimates.

## Methods

### Literature search and data extracted

We conducted a systematic search to identify published literature quantifying health impacts of air pollution and climate change. We searched five databases: PubMed, ProQuest Central, Science Direct, Scopus, and Web of Science. Search criteria were: (1) key words: climate change, global warming, future, air, O_3_, particles, PM, mortality, and health; (2) studies published between 2000 and 2011; and (3) only peer-review journal articles, government reports, and conference proceedings. We also searched manually for relevant references in articles found. Based on the search criteria, 14 studies were included in this review (Table 1). One study each was published in 2001, 2004, 2006 and 2007, three studies in 2008 and seven studies during 2009–2011. The majority were peer-reviewed journal articles except two studies, which were a proceedings paper (Cope et al. 2011a) and a government report (Anderson et al. 2001). Although the focus of this review was on quantitative health impact estimations, we also included two studies (Anderson et al. 2001; Casimiro et al. 2006) in which future projections were not expressed as quantitative changes in health risk. Rather, the future trends were predicted descriptively based on mixed quantitative and qualitative analyses on future climate, air quality and health impacts. The approaches applied in these two studies are referred to as semi-quantitative in this review.

**Table 1:** Study locations, baseline and projected periods, and health effects quantified

Reference	Study location	Baseline (B) and projected (P) period	Health effect
Anderson et al. 2001	British Isles	B: 1990	O_3_ and PM_10_-related to unspecified health outcomes
		P: decades in twenty-first century	
Bell et al. 2007	50 US cities	B: 1993–1997	O_3_-related non-accidental, cardiovascular and respiratory mortality; hospital admissions for COPD; respiratory and asthma
		P: 2053–2057	
Casimiro et al. 2006	Lisbon, Portugal	B: 1990s	NO_2_ and O_3_ -related to unspecified health outcomes
		P: 2020s and 2050s	
Chang et al. 2010	19 US communities	B: 2000	O_3_-related premature mortality
		P: 2041–2050	
Cheng et al. 2008	4 Canadian cities	B: 1981–2000	Extreme temperatures, CO, O_3_, NO_2_, SO_2_ and SP-related non-traumatic mortality
		P: 2040–2059 and 2070–2080	
Cope et al. 2011a	Sydney region	B: 1996–2005	O_3_-related respiratory hospital admissions
		P: 2021–2030 and 2051–2060	
Doherty et al. 2009	15 UK conurbations	B: 2000	Heat and O_3_-related premature mortality
		P: 2020–2030	
Jackson et al. 2010	2 counties, Washington State	B: 1997–2006	Heat and O_3_-related non-traumatic and cardiopulmonary mortality
		P: 2045–2054	
Jacobson 2008	World and the US	Comparing present days with preindustrial period	O_3_-related mortality; hospitalisation and emergency-room visits; PM_2.5_-related mortality; Non-methane VOCs-related cancer
Knowlton et al. 2008	New York region	B: 1990s	Heat and O_3_-related acute non-accidental mortality
		P: 2020s, 2050s, and 2080s	
Knowlton et al. 2004	31 New York counties	B: 1990s	O_3_-related all internal causes mortality
		P: 2050s	
Selin et al. 2009	16 world regions	B: 1999–2001	O_3_-related mortality; respiratory hospital admissions; respiratory symptom and minor restricted activity days; asthma; bronchodilator usage and lower respiratory symptoms
		P: 2049–2051	
Sheffield et al. 2011	14 New York counties	B: 1990s	O_3_-related childhood asthma
		P: 2020s	
Tagaris et al. 2009	United States	B: 2001	O_3_ and PM_2.5_-related premature mortality; respiratory and cardiovascular hospital admissions; acute respiratory symptoms; respiratory emergency room visits; school loss days
		P: 2050	

*CO* Carbon monoxide, *COPD* chronic obstructive pulmonary disease, *NO*_*2*_ nitrogen dioxide, *O*_*3*_ ozone, *PM*_*2.5*_ particulate matter with diameter 2.5 μm or less, *PM*_*10*_ particulate matter with diameter 10 μm or less, *SO*_*2*_ sulphur dioxide, *SP* suspended particles, *VOCs* volatile organic compounds

In each of the 14 studies, we examined its design, methods and, if available, results of sensitivity analyses to test associated uncertainties. Aspects of the study design we considered included study location, reference and projected time periods, and health effects (Table 1). Models and their corresponding scenarios applied to project climate, air quality and health impacts are listed in Table 2. A basic method commonly applied in all the reviewed papers is introduced in the following section on “General approach to quantifying health impacts associated with climate change” , followed by a section on “Approaches to quantifying the health impacts associated with air pollution and climate change”, which briefly provides the setting, methods and scope of each study. A “Discussion” then compares the strengths and weaknesses of individual studies relative to the optimal approaches recommended for this research area.

**Table 2:** Climate, air quality and health models and their scenarios of the reviewed studies

Reference	Climate projection		Air quality projection		Health impact projection	
	Model^a^	Scenario	Model^a^	Scenario	Model	Scenario
Anderson et al. 2001	GCM - HadCM3	IS92a	Historical air pollution episodes	NA	Concentration-response function	NA
Bell et al. 2007	GCM - GISS	SRES A2	CMAQ with SMOKE	Constant emissions	Concentration-response function	Population and age structure constant
	RCM - MM5					
Casimiro et al. 2006	GCM - HadCM2	CO_2_ concentrations two times larger than present	Historical air pollution episodes	NA	NA	NA
	RCM - PROMES and HadRM2					
Chang et al. 2010	GCM - CGCM3	SRES A2	Statistical prediction models	Constant emissions	Concentration-response function	Population and age structure constant
	RCM - Canadian RCM					
Cheng et al. 2008	GCM - CGCMs, US GFDL- R30 with statistical downscaling and synoptic weather typing	IS92a, SRES A2 and B2	Within-weather-type historical simulation models	(1) Constant emissions; (2) decreasing 20 % and (3) increasing 20 %	Within-weather group mortality prediction models	Population and age structure constant
Cope et al. 2011a	GCM - CSIRO Mk3	SRES A2	TAPM-CTM	(1) Constant emissions; and (2) decreasing 40 % and 70 % of O_3_ precursors	Concentration-response function	Population and age structure constant. 60 ppb O_3_ threshold
	RCM - CCAM					
Doherty et al. 2009	11 coupled global chemistry-climate models and 15 global chemistry models simulated by numerical weather prediction data				Concentration-response function	NA
Jackson et al. 2010	GCM - PCM	SRES A2	Global - MOZART-Regional - CMAQ with SMOKE	O_3_ precursors based on the EPA Economic Growth Analysis System and land-use models	Concentration-response function	Projected population and age structure for 2025
	RCM - MM5					
Jacobson 2008	GATOR - GCMOM	(1) CO_2_ at present-day conditions; and (2) CO_2_ at the preindustrial period	SMVGEAR II	NA	Concentration-response function	35 ppb O_3_ threshold
Knowlton et al. 2008	GCM - GISS	SRES A2	CMAQ with SMOKE	Constant emissions	Joint concentration response function of temperature and O_3_	Population and age structure constant
	RCM - MM5					
Knowlton et al. 2004	GCM - GISS	SRES A2	CMAQ with SMOKE	(1) Constant emissions; and (2) growth in O_3_ precursor emissions as identified in SRES A2	Concentration-response function	Population and age structure constant
	RCM - MM5					
Selin et al. 2009	GCM - GISS	SRES A1B	GEOS Chem	(1) Constant emissions; and (2) 2050 emissions	Concentration-response function	Population in 2050
Sheffield et al. 2011	GCM - GISS	SRES A2	CMAQ with SMOKE	Constant emissions	Concentration-response function	Population and age structure constant
	RCM - MM5					
Tagaris et al. 2009	GCM - GISS	SRES A1B	CMAQ with SMOKE	Constant emissions	Concentration-response function	Population and age structure constant
	RCM - MM5					

^a^Model names in full are available online (Annex 1)

*CO*_*2*_ Carbon dioxide, *EPA* Environmental Protection Agency, *GCM* general circulation model, *O*_*3*_ ozone, *ppb* part per billion, *RCM* regional climate model

### General approach to quantifying health impacts associated with climate change

All papers reviewed applied a common basic method as outlined by Campbell-Lendrum and Woodruff (2007). This method involves two main elements: (1) using historical records to measure the effect of climate variation on health; and (2) applying known, or estimated, relationships to projected climate at a chosen future time. The first element involves developing a concentration–response function for each health outcome that is believed to be sensitive to weather and climate. This aim can be achieved via time-series studies. For the second, the International Panel on Climate change (IPCC) has developed a series of emission storylines and scenario families, referred to as Special Report on Emissions Scenarios (SRES). These scenarios are based on different population growth schemes, economic conditions, social development, progress in technology development and transfer among regions (IPCC 2000).

The two elements described above are sufficient if a relative percent change in the health outcome of interest is to be estimated. However, if a more complete picture of impacts on population health is the goal of the quantification, the information from the two elements needs to be linked further with the baseline mortality or morbidity rate and the population size. The standard formula generally applied in the studies we reviewed (Chang et al. 2010; Jackson et al. 2010; Jacobson 2008; Knowlton et al. 2004, 2008; Selin et al. 2009; Sheffield et al. 2011; Tagaris et al. 2009) is given below.

1 $$ \varDelta H=R*\left( {{e^{{\beta *\varDelta C}}}-1} \right)*Pop $$

where *∆H* is the change in the health outcome of interest resulting from changes in an environmental factor, *R* is the baseline mortality or morbidity rate, *ΔC* is the estimated change in an environmental factor, *β* is the log relative risk associated with a change in exposure to the environmental factor, and *Pop* is the exposed population in the period and location of interest. From the point of view of future projections, different assumptions can be made about future rates of health endpoints *R* and populations *Pop*.

### Approaches to quantifying the health impacts associated with air pollution and climate change

**Fig. 1:**
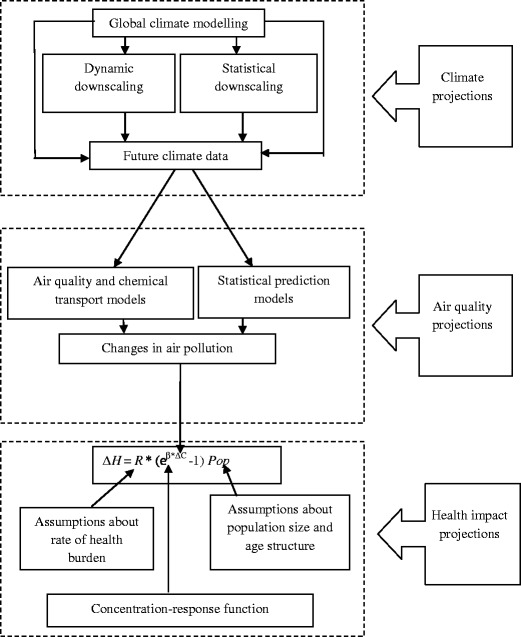
Major steps for projecting health impacts associated with air pollution and climate change. *∆H* Change in health outcome resulting from changes in air pollution exposure, *R* baseline annual mortality or morbidity rate, *β* log relative risk associated with a unit change in air pollution exposure, *ΔC* estimated change in air pollution concentration, *Pop* future exposed population

Studies attempting to estimate the health impacts, although sharing the same basis of the projections, have used different methods, each tailored to meet their goals and local context. Here we briefly summarise methods applied in individual studies that generally involve climate, air quality and health impact projections as depicted in Fig. 1. The output from a climate projection is coupled with an AQM to predict future air quality. The predicted change in air pollution is then used with a concentration-response function for predicting health impacts.

Two studies in this review focussed on estimations of the health impact associated with air pollution and climate change at the global scale. Jacobson (2008) compared mortality and morbidity due to exposure to O_3_, PM_2.5_ (PM with diameter 2.5 μm or less) and non-methane VOCs between present-day with preindustrial periods in which a near-surface temperature difference is 1.07 K. A general circulation model (GCM), GATOR-GCMOM, was nested with a box chemistry model, SMVGEAR II, to estimate differences of the pollutants between the two periods in this study. With the estimated changes of air pollutants, a concentration-response function for each pollutant derived from the literature was further applied to quantify the health impacts assuming a 35 ppb O_3_ threshold and zero thresholds for the other pollutants. The other study (Selin et al. 2009) examined relative changes in health impacts and the associated costs due to potential increased O_3_ for the period 2049–2051 in 16 world regions. Greenhouse gas (GHG) emissions based on the IPCC SRES A1B scenario were used to simulate a GCM, GISS GCM3. Projected climate was input to a global atmospheric and chemical transport model, GEOS-Chem, for estimating baseline and future O_3_ concentrations. Emissions of O_3_ precursors were held fixed at the stage of O_3_ projection. The health impact was quantified by using concentration-response functions gathered from a collection of original time-series and meta-analysis studies.

This research area has been more active in North America than the other regions. Knowlton et al. (2004) pioneered estimating future mortality due to O_3_ under a changing climate at the regional level in Metropolitan New York. To achieve the regional projection of climate and O_3_, a GCM, GISS, forced by IPCC SRES A2 was linked with a regional climate model (RCM), MM5, prior to integrating future simulated climate to a chemical transport model, CMAQ. The projections for the 2050s were compared with the reference period, 1990s. The projected O_3_ concentrations were applied to assess relative changes in the mortality based on a concentration-response function that was pooled from seven epidemiological studies with an assumption of a constant population and mortality rate over time. The same projection method was also employed in two other studies assessing impacts of joint exposure to heat and O_3_ on mortality risk (Knowlton et al. 2008) and O_3_-related childhood asthma (Sheffield et al. 2011) in the same location. This method was again applied in the study of Bell et al. (2007) to quantify relative changes in O_3_ concentrations and related mortality and hospital admissions between the reference period, 1993–1997, and a future period, 2053–2057, in 50 US eastern cities. Tagaris et al. (2009) modified this method slightly by using IPCC SRES A1B to drive the GISS global model and expanding the projection to assess future PM_2.5_ concentrations and associated health effects across the United States. Similarly, a slightly modified framework of climate and O_3_ modelling system from the studies above was used by Jackson et al. (2010), who downscaled a GCM, PCM, and a global chemistry model, MOZART-2, to the MM5 regional climate model and the regional chemistry model CMAQ for projecting O_3_ related-mortality at mid-century in two counties of Washington State. The O_3_ projection was based on an assumption of changes in O_3_ precursors according to future economic growth. In that study, future population growth was factored in with the use of population estimates for the years 2005–2030.

Chang et al. (2010) and Cheng et al. (2008) employed different methods from the other studies in the North American region when predicting health impacts from future changes in air pollution and climate. Chang et al. (2010) projected O_3_ levels in southeastern US in the 2040s by using the regional climate output from a coupled Canadian global-regional climate modelling system driven by IPCC SRES A2 scenario to fit a statistical prediction model. Thereafter a concentration-response function was obtained from a multi-city study to project mortality attributed to the future change in O_3_ assuming population and age structure held fixed at the baseline period (2000). Cheng et al. (2008) applied a synoptic weather-typing approach to predict impacts of future extreme temperatures and air pollution on mortality in two time windows (2040–2059 and 2070–2089) in four cities in south-central Canada. To predict climate and air pollution, output from an ensemble of multiple GCMs driven by three IPCC scenarios was downscaled statistically before being linked with a statistical prediction function corresponding to each meteorological and air pollution related-weather type. The prediction functions were developed through grouping daily historical observations that caused high air pollution levels and temperatures. Predicted air pollution and weather of each weather type were then used as predictors in a within-weather-group mortality prediction model. This study projected the impacts based on three future scenarios of air pollution emissions including the baseline during (1981–2000), 20 % higher and lower of the emissions than the baseline.

Regional projections of climate change impacts on health associated with air pollution have also been undertaken in some European countries and in Australia. Doherty et al. (2009) simulated O_3_ concentrations and temperatures across the UK by using an ensemble of coupled global climate-chemistry models for the baseline period (1993–2003) with likely three pollution emissions scenarios. The projected information was combined with risk estimates for these two environmental factors in 15 conurbations evaluated during the baseline period to project future mortality for 2030. In Australia, Cope et al. (2011a) projected relative changes in hospital admissions attributable to O_3_ exposure in Sydney Region between 1996 and 2005 and two future periods, 2021–2030 and 2051–2060. A chemical transport model, TAPM-CTM, was linked with a coupled global-regional climate modelling system forced by IPCC SRES A2 to predict meteorological and pollution fields at a 3-km grid spacing over the area of interest. When projecting the health effects, this study assumed constant anthropogenic O_3_ precursors emissions and population over time.

Projections made by Anderson et al. (2001) and Casimiro et al. (2006) can be described as semi-quantitative approaches. Anderson et al. (2001) projected the future occurrence of summer and winter air pollution episodes out to 2100 on the basis of changes in weather variables under an IPCC business-as-usual scenario, IS92a, along with possible trends in pollutant emissions. The predicted future occurrence of air pollution episodes for the British Isles was considered together with a baseline health estimate of PM_10_ (PM with diameter 10 μm or less) and surface O_3_ to describe future trends of the health impacts. Casimiro et al. (2006) examined recorded meteorological conditions during the 1990s and associated them with pollutant episodes for NO_2_ and surface O_3_ in Lisbon, Portugal. Climate output from two RCMs driven by an anticipated future CO_2_ emissions scenario for two time periods, 2020s and 2050s, was used and assessed possible NO_2_ and O_3_ air pollution episodes. Despite a lack of baseline data on health burdens associated with air pollution, the study was able to qualitatively estimate potential changes in health outcomes attributable to NO_2_ and surface O_3_.

## Results and discussion

In this section, we analyse and discuss strengths and weaknesses of the methods described above for each stage of the projections undertaken in the 14 studies. We also identify how these studies handled uncertainty, which is a significant issue when projecting future impacts of climate change.

### Climate projections

At this stage of the projection, a number of procedures have been recommended to cope with model and GHGs emissions uncertainties. It is widely recognised in climate science that one approach to address climate model uncertainty is to use an ensemble of models (Bader et al. 2008; Meehl et al. 2007). However, this strategy brings in a requirement of massive computational resources. Practically, therefore, among the studies we examined, only two studies were able to implement the model ensemble strategy (Doherty et al. 2009; Cheng et al. 2008). The use of global scale projections only, without exploiting additional resources for generating fine-resolution climate information, was the key factor that made it possible for Doherty et al. (2009) to use an ensemble of 26 global atmospheric chemistry models. The remaining studies were based on the use of a single GCM following by (for most studies) a dynamic downscaling approach, which is also computationally expensive. In the reviewed studies, it is likely that the process for selecting a single GCM was based primarily on the capacity of an individual GCM to best reproduce the climatology in the area of interest. For example, Bell et al. (2007), Knowlton et al. (2008) and Tagaris et al. (2009) used GISS—a GCM developed by the US Goddard Institute for Space Studies—in their research to project the health impacts of American populations. Likewise HadCM3—a GCM developed by a UK institution—was used to project health impacts in Britain and Portugal (Anderson et al. 2001; Casimiro et al. 2006). The projections made using these single GCMs, and the associated uncertainty will be best quantified for the study regions, where the output from the GCMs used was verified against historical observations. In any event, the use of projections from a single GCM has the potential to introduce bias, due to not accounting for uncertainty related to climate model physics. In an assessment of heat-related mortality from climate change in six cities, model bias was found to be larger than the uncertainties associated with future GHGs scenarios and downscaling (Gosling et al. 2012). Therefore it is strongly recommended that ensembles of models—‘multi-model ensembles’ or ‘perturbed physics ensembles’—should be used for projection studies where practical (IPCC 2007). Although Cheng et al. (2008) considered a regional impact, the authors were able to include projections from three GCMs. This is because this study applied regression-based statistical methods, which are more computationally efficient compared to the dynamic approach, in downscaling the GCM.

GCM climate change simulations are forced by a GHG emissions scenario. Again, using multiple future GHG emissions scenarios is the favoured approach to address GHG emissions uncertainty, particularly when making projections close to the end of the century where the emissions have a greater degree of uncertainty (Carter et al. 2007). However, this approach is certainly offset by the computational resources required to run the same model multiple times. Based on the studies we reviewed, only Cheng et al. (2008) and Knowlton et al. (2008) used the output of GCMs forced by more than one IPCC SRES scenario. These two studies purposely selected the IPCC SRES B2 as an alternative scenario representing a lower bound of GHG emissions in comparison with other IPCC SRES scenarios representing high GHG emissions. Within a medium timeframe, surprisingly, Knowlton et al. (2008) found a change in 2050s relative to 1990s for O_3_-related mortality estimated for New York City Metropolitan Region by using the B2 scenario, low emissions, 3 % larger than the A2 scenario, high emissions. Cheng et al. (2008), although stated making projections over two future time periods—2050s and 2080s—with the use of three different IPCC scenarios—IS92a, A2 and B2—did not provide detailed estimates of air pollution-related mortality for each scenario corresponding to each time period. In the same study, however, the authors found a small discrepancy of approximately 3 % for the period 2050s while comparing an average of percentage change across four Canadian cities for heat-related mortality driven by the A2 with B2 scenarios. The discrepancy became significantly greater (55 %) for the projected period 2080s. The results from these two studies suggest that only a small discrepancy of the health impacts between high and low GHG emissions scenarios can be expected if the time horizon for the projections is not beyond 2050s. This also suggests that relying on only a single GHG emissions scenario for studies aiming to project the health impacts when the focus of their projected time periods is before 2050s, therefore, would not significantly impact the projection results.

### Air quality projections

Following the generation of climate projections, meteorological fields are linked with an AQM. Similar to climate modelling, running multiple AQMs would ideally help detect uncertainties associated with diverse simulation processes and functions handled by different modelling systems. Likewise, projecting future air pollution under multiple likely pollution emissions scenarios would be a desirable way to handle uncertainties associated with future emissions.

As can be seen from Fig. 1, air quality projections can be achieved through either numerical or empirical modelling. In our review, most studies employed three-dimensional numerical chemical transport models (Bell et al. 2007; Cope et al. 2011a; Doherty et al. 2009; Jackson et al. 2010; Jacobson 2008; Knowlton et al. 2004, 2008; Selin et al. 2009; Sheffield et al. 2011; Tagaris et al. 2009). The ability of the numerical models to handle complex flows and non-linear chemical reactions of air pollution likely makes this approach more favourable. However, these advantages are offset by the high computational demands of such models. Consequently, this appears to limit the development of an AQM ensemble and thus becomes a constraint on exploring the uncertainty associated with AQMs. At the global scale, three of the reviewed studies employed single chemistry-climate models to simulate effects of the change in GHG emissions on global air quality (Jackson et al. 2010; Selin et al. 2009; Jacobson 2008). Only one study was able to simulate results of global surface O_3_ projections from an ensemble of atmospheric chemistry-climate models (Doherty et al. 2009). At the regional scale, all the studies projecting regional air quality were dependent on a single chemical transport model (Bell et al. 2007; Knowlton et al. 2004, 2008; Jackson et al. 2010; Sheffield et al. 2011; Cope et al. 2011a).

Application of a statistical prediction model for projecting future air quality was found in two studies (Cheng et al. 2008; Chang et al. 2010). Chang et al. (2010) developed a linear regression model relating three meteorological variables, namely total cloud cover, solar radiation, temperature, as predictors to the prediction of surface O_3_ concentrations during the year 2000 to forecast future surface O_3_. Although fundamentally Cheng et al. (2008) also developed statistical prediction models for predicting multiple air pollutants under global climate change, the approach in their study was different and more complex compared to the study of Chang et al. (2010). Due to the advantage of inexpensive computer resources of these empirical modelling schemes, similar to the statistical downscaling strategy, studies adopting them would have more opportunities to conduct analyses on uncertainty from various factors, including model uncertainty, contributing to changes in future air quality (Chang et al. 2010).

With regard to future pollution emissions scenarios, we found that the most common approach was to assume constant anthropogenic emissions. Less common, but applied in a few studies, for example the studies of Knowlton et al. (2004), Sheffield et al. (2011) and Selin et al. (2009), involved setting up emission projections consistent with the storylines identified in the IPCC SRES scenarios. Apart from these two approaches, some studies built an emissions scenario on the basis of trends in current technologies and other factors contributing to the emissions. For instance, Doherty et al. (2009) simulated surface O_3_ predictions based on three possible futures, one of which was a low emissions scenario assuming the implementation of currently available emissions control technologies (Dentener et al. 2005). Jackson et al. (2010) developed an emissions database to predict future air quality based on projections of economic growth and changes in land use for the period 2045–2054. Cope et al. (2011a) set up two future pollution emissions scenarios—assuming 40 % and 70 % reductions in O_3_ precursors emissions relative to the reference period—to evaluate the achievement of compliance with the current standards for O_3_ in the 2050s.

The literature we explored presented a variety of findings when air pollution emission projections were factored in to future air quality. Knowlton et al. (2004) and Sheffield et al.(2011) found a reduction in future O_3_ concentrations and health impacts alike when an increase in anthropogenic O_3_ precursor emissions was considered in conjunction with the impact of climate change. The authors explained that the reduction in O_3_ concentrations was likely to be associated with titration of O_3_ by higher concentrations of NO_x_. On the contrary, assuming the growth of anthropogenic O_3_ precursor emissions in the study of Doherty et al. (2009), projecting 2030 O_3_ for the entire UK, resulted in an additional O_3_ increase of 14 % relative to when the scenario of climate change alone with holding the emissions constant at the present level was considered. Likewise, Selin et al. (2009) estimated an average O_3_ increase across regions globally for 2050s of approximately 6 ppb under the assumption of increased O_3_ precursor emissions and a changing climate in addition to the isolated climate change impact. The diversity of findings from these studies may be due partly to different spatial resolutions in the projections and the areas projected, which led to differences in the magnitude of air pollution concentrations and corresponding density of populations exposed. However, they strongly demonstrated a large contribution of uncertainty associated with future air pollution emissions in air quality projections and a need to examine it in the projection process.

### Health impact projections

In quantifying the impacts of climate change on health, the critical steps that must be undertaken with care involve choosing concentration–response functions and considering likely future population scenarios. If chosen from the literature, concentration–response functions for major health outcomes should be from combining multiple well-designed epidemiological studies. It is also important to demonstrate uncertainties associated with a chosen concentration–response function through a sensitivity analysis as the chosen function will be certainly subject to change in the future, which may impact greatly on the projected results. To isolate the magnitude of impacts of climate change and future population demographics on air pollution-related health, varying these factors can be undertaken as part of sensitivity analyses.

Most studies we examined used a concentration–response function that was estimated based on a multi-city study or meta-analysis. Between these two approaches, using health effect estimates from multi-city studies is recommended due to the unavoidable publication biases associated with meta-analyses (Bell et al. 2005). Rather than using a single estimate, Bell et al. (2007) and Jacobson (2008) chose a set of estimates from a number of epidemiological studies for a given health endpoint. According to Bell et al. (2007), different concentration–response functions for a given health outcome could lead to a two-fold difference in percentage changes in health outcome induced by exposure to O_3_ and climate change for 2050s relative to the baseline period 1990s. Similarly Selin et al. (2009), with the application of Monte Carlo analysis to measure sensitivity to the concentration–response function for O_3_-related mortality, found that the limits of a 95 % probability interval differed by a factor of two. These findings indicate the importance of the choice of concentration-response function. This same issue was highlighted in a study of climate change and diarrheal disease that found that the choice of concentration–response function was more important than the choice of climate model (Kolstad and Johansson 2011).

Despite strong evidence of the health effects of long-term exposure to air pollution, only one study conducted by Jacobson (2008) explored the effect of increased exposure to non-methane VOCs on cancer in relation to climate change. This is an area that future studies should take into account to avoid misleading underestimations of the health impacts.

Alternatives to taking a concentration–response function from the literature are to estimate the function based on local data (Knowlton et al. 2008; Chang et al. 2010; Cheng et al. 2008). Despite using local data, Chang et al. (2010) applied the principle of multi-city study to identify a relationship of surface O_3_ and mortality through combining a relative risk estimate for each of the 17 US counties examined. Cheng et al. (2008) used a synoptic weather typing approach, to identify a within-weather-type health prediction function based on local data. Knowlton et al. (2008) had to rely on local data in estimating a joint relationship of O_3_ and temperature on mortality because such relationship had rarely been explored previously.

The frequency of extreme events involving simultaneous exposure of a population to high temperature and air pollution has been increasing since late twentieth century (Dear et al. 2005; Tong et al. 2010; Filleul et al. 2006). Such events are projected to be more frequent and intense in a warmer future climate (Clark et al. 2006). However, based on our review, this concern has gained modest attention. Among the existing studies, only one study attempted to estimate health impacts of interactions between temperature and air pollution in a future population (Knowlton et al. 2008). Other studies, although quantifying both the future health effects of heat and air pollution due to climate change, did not consider a combined effect of these two environmental risk factors (Doherty et al. 2009; Cheng et al. 2008; Jackson et al. 2010).

Other factors can also cause uncertainties at the stage of health impact projections. Some of these are associated with incomplete knowledge in estimating the concentration-response functions (Ren and Tong 2008). One example is the adverse health effects due to O_3_ exposure at low concentrations. Many studies have found linear relationships between exposure to criteria air pollutants and adverse health effects, with no threshold. However, a few have argued that PM and surface O_3_ exhibit non-linear exposure-response curves but with thresholds lower than current standards (Bell et al. 2006; Stylianou and Nicolich 2009). Different approaches to applying an O_3_ threshold in the health impact function found in our review reflect these on-going debates. Predominantly, the studies examined in this paper applied a linear concentration–response function of O_3_ exposure with zero thresholds (Anderson et al. 2001; Bell et al. 2007; Chang et al. 2010; Jackson et al. 2010; Knowlton et al. 2008; Selin et al. 2009; Sheffield et al. 2011). Conversely, the studies of Cope et al. (2011a) and Jacobson (2008) used non-zero thresholds. To deal with the uncertainty of the O_3_ threshold on health impact predictions, a sensitivity analysis could be conducted to determine how applying a threshold level would affect final health estimates. For example, Knowlton et al. (2004) included a 20-ppb O_3_ threshold in one of their sensitivity analyses. With the threshold assumption, a slight larger climate-driven increase in O_3_-related mortality was found.

Estimating the future health burden in a particular location requires making assumptions about population demographics, and baseline mortality or morbidity rates of interest. While most studies we examined held these factors constant over the projected time period, a few studies chose to simultaneously bring population growth to play in the quantification, so that a joint effect of future changes in population size and the impacts of climate change was determined explicitly (Jackson et al. 2010; Selin et al. 2009). This is important as the uncertainty originating from future population growth has been identified to have the greatest influence, among other uncertainties associated with health impact projections, on the results of projecting the health impacts (Knowlton et al. 2004; Sheffield et al. 2011). As clearly shown from a sensitivity analysis conducted by Knowlton et al. (2004), which took into account population growth corresponding to the IPCC SRES A2 scenario, an estimate of O_3_-related mortality for the period 2050 was increased by more than 50 % relative to considering just the impacts of climate change or the combined impacts of climate change and O_3_ precursor emissions. Similarly, although to a lesser extent, Sheffield et al. (2011) found an additional 3.3 % of future O_3_-related respiratory emergency department visits in children for 2020s when an age-specific future population projection was taken into account together with the effect of climate change. Although it is well known that the health risks are higher among the elderly and children, this study was the only one, among the others we reviewed, that applied an age-specific concentration-response function and included information not only on the growing population size but also on future age demographics. Considering changes in size and age demographics are both equally imperative as many countries, even some developing countries, are transitioning to an aging society.

## Conclusions

Estimating the health impacts of air pollution and climate change involves linking climate, air quality and health projections. This interdisciplinary area of research is still at an early stage in its development as can be seen from the limited number (only 14) of studies found from the literature search. Additionally, O_3_ has been the primary focus of this research, with only a limited amount of work done on other pollutants such as PM. Although facing technical challenges, recent studies have developed methods that are applicable to different conditions and can improve reliability and transparency of the prediction results. The following is a summary of approaches commonly applied and a recommendation of methods for providing credible health estimates based on pros and cons of the studies we examined. Areas that will help improve the health impact estimation, but have received minimal or no attention yet, are identified at the end of this section.

In the studies we reviewed, climate projections were based mostly on a single GCM driven by a single IPCC GHG emissions scenario such as A1B or A2. When a regional projection was undertaken, a single RCM was used for dynamic downscaling. Clearly, a high demand for computational resources in running these numerical models results in reduced opportunities to investigate the variability of climate models and future GHG emissions, particularly when output at higher resolution for projecting regional climate is required. To overcome this challenge, the statistical downscaling technique, particularly when used in combination with statistical prediction models in the later stage of projecting air pollution, offers an alternative approach with computational efficiency. This approach allows flexibility in running a GCM model ensemble and comparing multiple future GHG emissions scenarios, a critical step in making projections close to the end of the century, while exploring other future uncertainties. In relation to the choice of future GHG emissions scenarios, we recommend the use of the IPCC SRES business-as-usual scenario such as A2 or A1B if the time horizon for the projections is not beyond 2050s. If the coverage of a projected time span goes beyond 2050s, at least two scenarios representing low and high emissions should be compared. However, these recommendations on the choice of IPCC scenarios might be subject to change due to development of four new key scenarios of future GHG emissions that will replace the SRES (Moss et al. 2010).

For air quality projections, the common approach was to integrate the projected climate information into air quality numerical models while holding future air pollution emissions constant. Taking advantage of advances in downscaling techniques, we found an increasing trend towards the use of regional air quality projections. This effort should be continued as it is critical to long-term air quality management. Taking into account different air pollution emissions scenarios should, from our perspective, be part of the air quality projections. This is particularly important in highly polluted areas with rapidly growing trends in economic and industrial development.

The most common approach in the health impact projections involved taking a concentration-response function from published literature and assuming no change in the current population and background health outcome rates. With respect to the choice of concentration–response function, we recommend the use of a relative risk function derived from a multi-site study. We also recommend conducting a sensitivity analysis to explore uncertainty from different concentration–response functions. In terms of population scenarios, we recommend consideration of changes in future demographics, both size and age structures. In case the full exercise of quantifying the impacts of climate change on air quality and health at local scale cannot be undertaken, at the very least, a semi-quantitative approach is recommended.

Three topics with regard to quantifying the health impacts should be priorities for future research. The first priority is about estimating future health impacts of extreme air pollution events including forest fires and dust storms. It is clear that there are strong associations of major forest fires and dust storms with a range of health outcomes (Analitis et al. 2012; Hashizume et al. 2010). There is also a possibility that climate change may cause increases in the occurrence of these extreme air pollutions as the century progresses (Flannigan et al. 2009; Aldersley et al. 2011). Although still premature in the current research, some progress has been made in projecting future air pollution extreme events in particular forest fires (IPCC 2007; Carvalho et al. 2011). Therefore, future studies should consider incorporating the health impacts of extreme air pollution events in the quantification. The second priority concerns behavioural adaptation of populations to cope with a warmer climate, which may modify exposure to air pollution. In projecting future temperature-related health effects, these changes have been important factors commonly taken into consideration (Kinney et al. 2008; Gosling et al. 2009). Some of them, such as opening windows and spending more time outdoors, have potential to alter not only the health risks of heat but also health impacts of air pollution (Barnett and Hansen 2009). The last priority is to investigate interactions between temperature and air pollution. Although still limited to date, we did find one study in this review that attempted to factor in the combined effects in the projection. Further advances in this research area should take advantage of a growing body of empirical relationships derived from epidemiological studies of the interactive effects (Qian et al. 2008; Ren et al. 2011).

## Electronic supplementary material

Below is the link to the electronic supplementary material.

ESM 1 (DOCX 12 kb)
