# Supplementary material for: Quantifying the health impacts of air pollution under a changing climate—a review of approaches and methodology
Source: Int J Biometeorol. 2013 Jan 25;58(2):149–60. doi: 10.1007/s00484-012-0625-8 (PMC3936128; doi:10.1007/s00484-012-0625-8)
Supplement: Supplementary file 1 — (DOCX 12 kb) [file 484_2012_625_MOESM1_ESM.docx]

Annex 1. Full names of climate and air quality models stated in Table 1b

| Acronym | Full name |
| --- | --- |
| AGCM3 | The Canadian Centre for Climate Modeling and Analysis third generation atmospheric general circulation model |
| CCAM | Cubic Conformal Atmospheric Model |
| CMAQ | Community Multiscale Air Quality model |
| CSIRO Mk 3.0 | Global climate modeling system from Commonwealth Scientific and Industrial Research Organisation, Australia |
| GATOR-GCMOM | Gas, Aerosol, Transport, Radiation, General Circulation, Mesoscale, and Ocean Model |
| GEOS Chem | Global 3-D chemical transport model for atmospheric composition driven by meteorological input from the Goddard Earth Observing System (GEOS) of the [NASA Global Modeling and Assimilation Office](http://gmao.gsfc.nasa.gov) |
| GFDL | Geophysical Fluid Dynamics Laboratory |
| GISS | Goddard Institute for Space Studies |
| HadCM2 | Hadley Centre Climate Model |
| HadRM2 | A regional climate model for Europe developed at the Hadley Centre, UK |
| MM5 | Mesoscale Model 5 from Penn State/National Centre for Atmospheric Research |
| MOZART 2 | Model for Ozone and Related Chemical Tracers version 2.4 |
| PCM | Parallel Climate Model |
| PROMES | A regional climate model for the Iberian Peninsula developed at the Universidad Complutense de Madrid |
| SMOKE | Sprase Matrix Operator Kernel Emissions Modeling System |
| SMVGEAR II | Sparse Matrix, Vectorized Gear code |
| STOCHEM | UK Meteorological Office Global Three-Dimensional Lagrangian Chemistry Model |
| TAPM –CTM | The Atmospheric Pollution Model - Chemical Transport Model |
